# Supplementary material for: Complete Mitochondrial Genomes of Two Water Mite Species in the Family Sperchontidae (Acari: Hydrachnidiae): Characterization and Phylogenetic Implications
Source: Genes (Basel). 2025 Oct 19;16(10):1236. doi: 10.3390/genes16101236 (PMC12563358; doi:10.3390/genes16101236)
Supplement: Supplementary file 1 [file genes-16-01236-s001.zip › Table S1.pdf]

Table S1 Mitochondrial genome organization of *Sperchon plumifer*

Genes	Strand	Position	Length	ovl/nc	Start codon	Stop codon	anticodon
cox1	J	1-1545	1545	2	ATG	TAA	
cox2	J	1545-2208	664	-1	ATG	T	
trnK	J	2209-2268	60	0			CUU
trnD	J	2269-2328	60	0			GUC
atp8	J	2329-2481	153	0	ATC	TAA	
atp6	J	2475-3137	663	-7	ATG	TAA	
cox3	J	3137-3917	781	-1	ATG	T	
trnE	J	3918-3973	56	0			UUC
trnG	J	3974-4023	50	0			UCC
nad5	N	4024-5692	1669	0	TTG	T	
nad4L	N	5711-5959	249	18	ATA	TAA	
trnA	J	5996-6044	49	36			UGC
nad3	J	6060-6389	330	15	ATA	TAA	
trnF	N	6391-6445	55	1			GAA
trnT	J	6452-6505	54	6			UGU
trnY	N	6501-6559	59	-5			GUA
trnN	J	6562-6620	59	2			GUU
trnL1	N	6616-6673	58	-5			UAG
CR	J	6674-7481	808	0			
trnS1	J	7482-7531	50	0			GCU
trnH	N	7581-7634	54	49			GUG
nad4	N	7685-8986	1302	50	ATG	TAA	
trnR	N	8986-9049	64	-1			UCG
trnV	J	9043-9092	50	-7			UAC
nad6	J	9093-9536	444	0	ATA	TAA	
cob	J	9536-10633	1098	-1	ATG	TAA	
trnS2	J	10635-10686	52	1			UGA
12s	N	11349-10691	659	4			
trnP	N	11350-11405	56	0			UGG
nad1	N	11406-12299	894	0	TTG	TAG	
trnL2	N	12300-12357	58	0			UAA
16S	N	12354-13382	1029	-4			
trnQ	N	13383-13440	58	0			UUG
trnI	J	13441-13495	55	0			GAU
trnM	J	13536-13596	61	40			CAU
nad2	J	13597-14547	951	0	ATA	TAA	
trnW	J	14546-14600	55	-2			UCA
trnC	N	14592-14644	53	-9			GCA
