# Supplementary material for: Complete Mitochondrial Genomes of Two Water Mite Species in the Family Sperchontidae (Acari: Hydrachnidiae): Characterization and Phylogenetic Implications
Source: Genes (Basel). 2025 Oct 19;16(10):1236. doi: 10.3390/genes16101236 (PMC12563358; doi:10.3390/genes16101236)
Supplement: Supplementary file 1 [file genes-16-01236-s001.zip › Table S2.pdf]

Table S2 Mitochondrial genome organization of *Sperchon* sp.

Genes	Strand	Position	Length	ovl/nc	Start codon	Stop codon	anticodon
cox1	J	1-1545	1545	1	ATG	TAA	
cox2	J	1545-2208	664	-1	ATG	T	
trnK	J	2209-2269	61	0			CUU
trnD	J	2269-2329	61	-1			GUC
atp8	J	2330-2482	153	0	ATT	TAA	
atp6	J	2476-3138	663	-7	ATG	TAA	
cox3	J	3138-3918	781	-1	ATG	T	
trnE	J	3919-3974	56	0			UUC
trnA	J	3992-4041	50	17			UGC
nad3	J	4057-4386	330	15	ATA	TAA	
trnT	J	4393-4447	55	6			UGU
trnN	J	4476-4535	60	28			GUU
CR1	J	4536-4963	428	0			
trnY	N	4964-5023	60	0			GUA
CR2	J	5024-5464	441	0			
trnS1	J	5465-5515	51	0			GCU
trnG	J	5527-5576	50	11			UCC
nad5	N	5557-7245	1669	0	TTG	T	
nad4l	N	7263-7547	285	17	ATG	TAG	
trnF	N	7564-7619	56	16			GAA
trnL1	N	7636-7694	59	16			UAG
trnH	N	7713-7766	54	18			GUG
nad4	N	7769-9070	1302	2	ATG	TAG	
trnR	N	9071-9121	51	0			UCG
trnV	J	9120-9169	50	-2			UAC
nad6	J	9170-9613	444	0	ATA	TAA	
cob	J	9613-10710	1098	-1	ATG	TAA	
trnS2	J	10714-10765	52	3			UGA
12s	N	10770-11429	660	4			
trnP	N	11430-11487	58	0			UGG
nad1	N	11488-12381	894	0	TTG	TAA	
trnL2	N	12382-12439	58	0			UAA
16S	N	12436-13458	1023	-4			
trnQ	N	13467-13522	56	8			UUG
trnI	J	13523-13575	53	0			GAU
trnM	J	13615-13675	61	39			CAU
nad2	J	13676-14626	951	0	ATA	TAA	
trnW	J	14625-14680	56	-2			UCA
trnC	N	14673-14723	51	-8			GCA
